# Monomeric C-reactive Protein as a Mediator of Chronic Inflammation in Obesity

**DOI:** 10.7759/cureus.108410

**Published:** 2026-05-07

**Authors:** Alina Pentek, Anca Bacârea

**Affiliations:** 1 Clinical Laboratory, County Emergency Clinical Hospital of Târgu Mureș, Târgu Mureș, ROU; 2 Department of Pathophysiology, George Emil Palade University of Medicine, Pharmacy, Science, and Technology of Târgu Mureș, Târgu Mureș, ROU

**Keywords:** adipose tissue, chronic inflammation, endothelial dysfunction, leptin resistance, macrophages, metabolic dysfunction, monomeric c-reactive protein, obesity, oxidative stress, platelets

## Abstract

Obesity is associated with persistent low-grade inflammation. This inflammation results from interactions between adipose tissue, immune cells, and metabolic pathways. High-sensitivity C-reactive protein (CRP) serves as a marker for systemic inflammation but does not evaluate local inflammatory processes. Recent findings indicate that the monomeric variant of CRP is biologically active and may contribute to inflammation in multiple tissues, including adipose tissue. This review examines the role of monomeric CRP (mCRP) as a mediator of ongoing inflammation in obesity and discusses the mechanisms through which it contributes to endothelial dysfunction, immune activation, and metabolic changes. We created a narrative review utilizing the PubMed electronic database. Relevant research on CRP isoforms, mCRP development, cellular targets, and their involvement in obesity-related inflammation was included and examined. mCRP forms locally under conditions such as oxidative stress, membrane injury, and cellular activation and may trigger proinflammatory effects in the endothelium, macrophages, platelets, and adipose tissue. mCRP may stimulate the endothelium, leading to heightened expression of adhesion molecules. This aids in leukocyte recruitment and reduces nitric oxide availability. mCRP may encourage macrophage polarization toward a proinflammatory type and enhance the formation of neutrophil extracellular structures. At the level of platelets, it has been shown to increase platelet aggregation and may aid in thrombus stabilization. Moreover, mCRP has been associated with increased reactive oxygen species production in experimental settings, creating a loop of inflammatory amplification. mCRP could disrupt leptin signaling and may contribute to leptin resistance. The conclusions of this review suggest that mCRP acts as an active mediator of chronic inflammation associated with obesity. It is more than a passive biomarker, as localized production may amplify tissue-level inflammatory effects. This may explain the disparity between systemic inflammatory indicators and ongoing vascular and metabolic inflammation. Additional studies are needed to standardize detection techniques and to define their clinical significance in risk assessment and treatment strategies.

## Introduction and background

Obesity is a chronic, multiorgan inflammatory condition that begins early with the progressive accumulation of adipose tissue and may precede the onset of overt metabolic disease [1-3]. This inflammatory state directly contributes to the development of insulin resistance, endothelial dysfunction, and increased cardiovascular risk [4-8]. Early metabolic changes can be identified using metabolic and inflammatory indices, even in young populations [3,9,10].

Inflammation in obesity extends beyond adipose tissue and involves the liver, skeletal muscle, pancreas, and vascular endothelium, contributing to the development of chronic noncommunicable diseases [1,3,6,7].

High-sensitivity C-reactive protein (hsCRP) reflects the activation of the adipose tissue-interleukin (IL)-6-liver axis and is widely used as a marker of systemic inflammation and cardiometabolic risk, correlating with adipometric parameters and metabolic syndrome [6,7,11]. However, standard CRP assays do not distinguish between structural isoforms and do not provide information on the location or activity of tissue-level inflammation, thereby limiting the identification of focal inflammatory processes in obesity [12,13].

Monomeric C-reactive protein (mCRP) is the biologically active form of CRP, generated locally in the inflammatory microenvironment through conformational conversion of the pentameric form. This process exposes proinflammatory binding sites and enables interactions with endothelial cells, platelets, and immune cells, activating signaling pathways involved in inflammation and vascular dysfunction. These effects involve integrin-mediated signaling and activation of intracellular pathways such as phosphatidylinositol 3-kinase-protein kinase B (PI3K-AKT) and nuclear factor kappa B (NF-κB), which promote the inflammatory response [12,14-16].

Available data indicate that mCRP accumulates in affected tissues and may more accurately reflect local inflammatory activity than total CRP [12,16]. Furthermore, circulating mCRP correlates with tissue expression, supporting its role as a link between local and systemic inflammation [17].

Despite the increased interest in CRP isoforms, the specific role of mCRP in obesity-associated inflammation remains poorly defined, particularly regarding its contribution to tissue inflammation and metabolic dysfunction [18].

The aim of this review is to investigate the role of mCRP as a mediator of ongoing inflammation in obesity and to examine the mechanisms through which it contributes to endothelial dysfunction and cardiometabolic risk [13,16,18].

## Review

Methods

A narrative review was conducted based on the literature identified in the PubMed database. Studies published between 2000 and 2025 were included. The search strategy used combinations of keywords such as “C-reactive protein”, “monomeric CRP”, “mCRP”, “obesity”, “inflammation”, “endothelial dysfunction”, and “adipose tissue”.

Studies were selected based on their relevance to CRP isoforms, mechanisms of mCRP formation, cellular targets, and their role in obesity-associated inflammation. Articles were prioritized based on mechanistic relevance. Experimental, observational, and review studies were included. Only articles published in English were considered.

Studies investigating mCRP in other inflammatory conditions, particularly cardiovascular and neurodegenerative diseases, were also included, as they provide mechanistic insights into mCRP formation, distribution, and biological effects at the tissue level. These mechanisms are considered relevant and transferable to chronic inflammation in obesity.

No formal quality assessment, statistical analysis, or meta-analytic methods were applied, consistent with the narrative design. Most of the available evidence derives from experimental models and cardiovascular pathology, while direct evidence on obesity remains limited. This review was conducted as a narrative synthesis and did not follow a formal PRISMA framework. No formal risk-of-bias assessment tools were used, and studies were selected based on relevance and scientific contribution rather than on quantitative criteria.

Isoforms and structure

C-reactive protein (CRP) exists in multiple structural isoforms that differ in their biological properties and relevance to inflammation, being present not only in its native pentameric form but also in several nonpentameric isoforms [19]. These include monomeric CRP (mCRP), intermediate forms (representing transitional states between pentameric C-reactive protein (pCRP) and mCRP), and other oligomeric structures, including trimeric and tetrameric CRP. These conformations can be identified using nonreducing techniques, including Western blotting, which preserve native structural organization [20-22].

Nonpentameric forms have been detected in various inflamed tissues, including the vascular wall, ischemic muscle, and injured brain tissue. Their presence correlates with systemic inflammatory processes and with metabolic and renal diseases, supporting their biological relevance beyond simple structural variants [20,22-24].

CRP circulates as a heterogeneous population of structural isoforms. Some modified forms associate with obesity independently of total circulating CRP levels, suggesting that molecular conformation may have greater biological relevance than overall concentration [24,25]. Multimeric isoforms, such as trimeric and tetrameric CRP, have been described especially in elderly individuals. Their presence suggests a link with chronic low-grade inflammation associated with aging, although a direct causal role remains unclear [20,26-28]. Under inflammatory conditions, pCRP can undergo conformational changes, generating bioactive isoforms such as mCRP and intermediate forms [22]. CRP is an acute-phase protein synthesized predominantly in the liver under the control of proinflammatory cytokines, especially IL-6 [28-30].

In circulation, CRP exists almost exclusively as pCRP, composed of five identical subunits arranged in a stable, calcium-dependent structure [23,31,32]. This form serves as a clinical biomarker of inflammation. Functionally, pCRP is relatively inert under physiological conditions and does not induce direct cellular activation in rigorous experimental models [15,33,34]. Its structural stability explains the absence of direct proinflammatory effects on endothelial or immune cells in the absence of specific local triggers [35].

Monomeric CRP (mCRP) represents the bioactive isoform generated at sites of inflammation. This process leads to loss of pentameric symmetry and exposure of neoepitopes that confer distinct biological properties. mCRP is structurally unstable, shows increased affinity for cell membranes, and exhibits significantly stronger proinflammatory activity in experimental models. It promotes endothelial activation, leukocyte recruitment, and amplification of local inflammatory responses [33,34].

Where and How Does mCRP Form?

mCRP forms locally through dissociation of pCRP on activated cellular membranes in a process dependent on lysophosphatidylcholine, oxidized phospholipids, and phospholipase A2 activation [14,36-38]. This conversion does not occur spontaneously in circulation but represents a strictly localized process dependent on the tissue microenvironment. Experimental and in vivo observations indicate that pCRP dissociation occurs on activated platelets, endothelial cells, and microparticles derived from inflammatory cells. A central mechanism involves phospholipase A2 activation and lysophosphatidylcholine exposure on activated membranes. These lipid alterations destabilize the pentameric structure and promote local mCRP accumulation within vascular lesions and atherosclerotic plaques [14,36]. On platelets, CRP monomerization depends on glycoprotein IIb-IIIa (GPIIb-IIIa) activation and occurs under physiological flow conditions. This process retains mCRP within platelet aggregates and amplifies local inflammation and thrombosis [39].

An additional phospholipid-independent mechanism has been described. CRP exhibits mechanosensitive properties, and increased shear stress may directly induce pCRP conversion to mCRP. This mechanism is clinically relevant in conditions involving abnormal hemodynamic flow and expands the paradigm of CRP activation beyond classical lipid-dependent inflammation [40].

Receptors and Cellular Targets of mCRP

In chronic inflammation, mCRP can exert effects on several cell types, such as endothelial cells, monocytes, neutrophils, platelets, and macrophages, as well as indirectly on adipocytes [14,34,37,38,41,42]. At the endothelium, mCRP has been shown to increase the production of adhesion molecules such as intercellular adhesion molecule 1 (ICAM-1), vascular cell adhesion molecule 1 (VCAM-1), and E-selectin (endothelial selectin), as well as proinflammatory cytokines such as IL-6 and tumor necrosis factor alpha (TNF-α) [34].

The interaction between mCRP and immune cells is induced by specific receptors. mCRP has been shown to activate the αvβ3 (alpha-v beta-3 integrin) and α4β1 (alpha-4 beta-1 integrin) integrins through the Arg-Gly-Asp binding motif site, activate the PI3K-AKT pathway, and induce monocyte chemotaxis. pCRP does not exhibit these interactions due to structural constraints, confirming the functional differences between the two forms [41].

At the endothelial progenitor cell level, mCRP and pCRP exert opposite effects. mCRP may induce a proinflammatory transcriptional program and alter its function, contributing to vascular dysfunction. pCRP has different or opposite effects [43]. In addition to local conversion from pCRP, mCRP may also be produced locally by macrophages, supporting a tissue-autonomous source of biologically active CRP [44-48].

mCRP can be transported by monocyte-derived exosomes. This mechanism allows the dissemination of the inflammatory signal to distant vascular territories without significant increases in serum CRP. This process explains the discrepancy between persistent local inflammation and the relatively modest hsCRP levels characteristic of chronic low-grade inflammation [44].

Obesity as a Model of Chronic Inflammation

In obesity, adipose tissue exceeds its metabolic role and becomes an active immune organ. Adipocyte hypertrophy with the creation of metabolic stress and lipid overload determines the attraction of macrophages with a proinflammatory phenotype and the initiation of inflammatory loops with their own amplification, which are associated with local hypoxia and adipocyte death [1,3-5,49,50]. The expansion of adipose mass exceeds the vascular adaptation capacity and causes local hypoxia [3,51,52].

The adipose tissue microenvironment in obesity is characterized by oxidative stress, endothelial activation, and alterations in biological surfaces [3,50,53]. Hypoxia induced by insufficient vascularization of expanded adipose tissue activates hypoxia-inducible factor 1 alpha and maintains a persistent proinflammatory profile, with increased IL-6, leptin, and other inflammatory adipokines [50,52]. These conditions create the biological framework necessary for the local activation of inflammatory mediators and the recruitment of immune cells to adipose tissue, thereby amplifying the local inflammatory response [53,54].

This environment favors the recruitment of proinflammatory M1-like macrophages, which progressively replace M2 macrophages [1,3,6,7,49]. Local production of IL-6, TNF-α, and monocyte chemoattractant protein-1 (MCP-1) increases [1,4,49,50,53]. Inflammatory resolution mechanisms become insufficient, and inflammation becomes chronic [3,7].

Inflammation is supported by several converging factors. Increased lipolysis from hypertrophied adipocytes releases free fatty acids that activate NF-κB and c-Jun N-terminal kinase (JNK) pathways and induce oxidative stress in adipocytes and immune cells [4,5,55]. Intestinal dysbiosis causes inflammation in obesity. Dietary imbalances, especially excess fat, alter the composition of the microbiota, with a reduction in beneficial bacteria and an increase in proinflammatory species rich in lipopolysaccharides (LPS) [46,56,57].

The integrity of the intestinal barrier is impaired by dysfunction of tight junction proteins, which leads to increased intestinal permeability. As a result, bacterial LPS enter the circulation, causing metabolic endotoxemia [58,59]. LPS activate toll-like receptor 4 and cluster of differentiation 14 receptors and induces the production of proinflammatory cytokines such as IL-1β, IL-6, and TNF-α. These maintain a state of low-grade systemic inflammation and promote insulin resistance [58-60].

Inflammation persists, and endothelial activation increases. Favorable conditions are created for local CRP activation and amplification of inflammatory signaling. mCRP may exert proinflammatory effects on the endothelium and immune cells and amplify local inflammation [14,15]. Thus, intestinal dysbiosis contributes to the initiation of inflammation and the formation of mCRP, supporting the maintenance of chronic inflammation in obesity [15,56,61,62].

Clinical biomarkers of inflammation in obesity

hsCRP reflects activation of the adipose tissue, IL-6, and liver axis, and is used for cardiovascular risk stratification. IL-6 and TNF-α indicate proinflammatory cytokine activation, the leptin/adiponectin ratio reflects adipokine imbalance, and fibrinogen suggests a proinflammatory and prothrombotic status [6,63-65]. These biomarkers predominantly reflect systemic inflammation and do not provide direct information about tissue-level inflammatory activity [3,7].

Through its association with activated endothelium, platelets, and immune cells, mCRP reflects active inflammation at the vascular and metabolic levels. This may explain why some patients with obesity exhibit increased cardiovascular risk despite normal or low hsCRP values, as local inflammatory processes may persist without a proportional increase in systemic CRP levels [14,25,66].

The mCRP/hsCRP ratio may provide an additional insight into the relationship between systemic inflammation and local inflammatory processes. hsCRP reflects circulating pCRP produced by the liver, while mCRP represents the bioactive form generated at sites of inflammation. A higher relative proportion of mCRP may indicate increased inflammatory activity that is not captured by hsCRP. This ratio is not yet standardized for clinical use and requires validation in prospective studies [14,25,66,67].

Mechanisms by Which mCRP Amplifies Inflammation in Obesity

**Figure 1:**
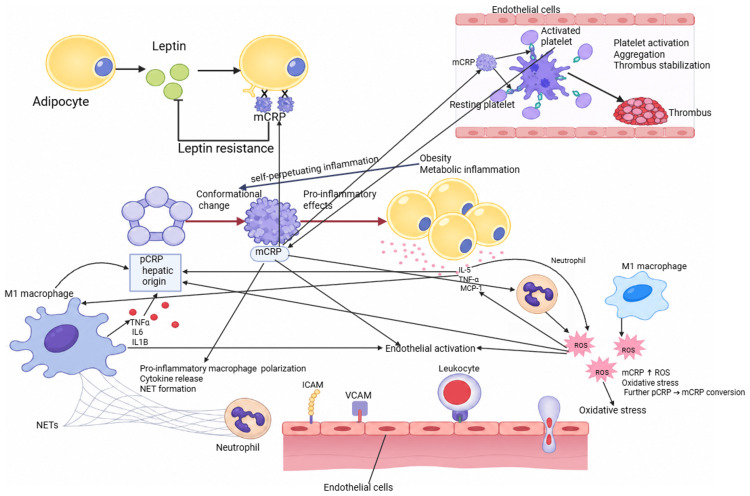
Mechanisms by which mCRP amplifies inflammation in obesity The figure illustrates the mechanisms by which mCRP amplifies inflammation in obesity and maintains a vicious inflammatory cycle. pCRP, produced in the liver, undergoes changes according to conditions of oxidative stress and local inflammation and is converted to mCRP. mCRP acts locally and initiates proinflammatory effects. At the level of adipose tissue, mCRP contributes to metabolic inflammation. Adipocytes release cytokines such as IL-6, TNF-α, and MCP-1, which favor the recruitment of immune cells and maintain chronic inflammation. In parallel, leptin resistance occurs, which worsens the metabolic imbalance. mCRP stimulates macrophages toward a proinflammatory M1 phenotype. They release cytokines and induce the formation of NETs by activating neutrophils. Neutrophils and macrophages produce reactive oxygen species, which increase oxidative stress and favor the further conversion of pCRP to mCRP. At the endothelial level, mCRP activates endothelial cells. The expression of adhesion molecules ICAM and VCAM increases, which allows leukocyte attachment and migration into the vascular wall. At the same time, mCRP activates platelets. This process leads to platelet aggregation and thrombus stabilization, increasing the risk of thrombotic events. All these mechanisms are interconnected and mutually amplify each other. The result is a persistent, self-perpetuating inflammation characteristic of obesity mCRP: monomeric C-reactive protein; pCRP: pentameric C-reactive protein; IL-6: interleukin 6; IL-5: interleukin 5; TNF-α: tumor necrosis factor-alpha; IL-1B: interleukin 1 beta; MCP-1: monocyte chemoattractant protein-1; NETs: neutrophil extracellular traps; ICAM: intercellular adhesion molecule; VCAM: vascular cell adhesion molecule; ROS: reactive oxygen species Image credit: This image was created by the authors using BioRender.com (BioRender, Toronto, Canada)

In obesity, persistent metabolic inflammation favors local monomerization of pCRP. mCRP becomes an active tissue mediator and amplifies inflammation through convergent effects on the endothelium, macrophages, platelets, and energy metabolism [14,66], as summarized in Figure 1.

Endothelium

mCRP induces endothelial activation through specific molecular pathways. It upregulates adhesion molecules such as ICAM-1, VCAM-1, and E-selectin and increases the production of proinflammatory cytokines, including IL-6 and TNF-α [34,41]. These effects are mediated in part by activation of integrin receptors, particularly αvβ3, followed by stimulation of the PI3K-AKT signaling pathway. This signaling cascade promotes a proinflammatory endothelial phenotype and enhances leukocyte adhesion and transendothelial migration [41].

mCRP also increases endothelial permeability and reduces nitric oxide bioavailability, contributing to impaired vasodilation and endothelial dysfunction [14,34]. In the context of obesity, where the endothelium is already exposed to chronic metabolic and inflammatory stress, these mechanisms may further amplify vascular inflammation and promote atheroinflammatory processes [3,6].

Macrophages

mCRP may promote macrophage activation and enhance their proinflammatory behavior, contributing to increased secretion of cytokines such as IL-6, TNF-α, and MCP-1 [14]. In obesity, macrophage polarization toward a proinflammatory M1 phenotype is driven by metabolic stress and activation of inflammatory pathways such as NF-κB and JNK, which sustain cytokine production and immune cell recruitment [1,4]. Activation of the NLR family pyrin domain containing 3 inflammasome further contributes to IL-1β and IL-18 production and maintenance of chronic inflammation. In this context, mCRP may amplify preexisting inflammatory pathways and contribute to the persistence of local inflammation [1,3].

mCRP also promotes monocyte adhesion, transmigration, and reactive oxygen species (ROS) generation, contributing to tissue injury and amplification of inflammatory signaling [14]. mCRP may contribute to neutrophil activation and the formation of extracellular structures, which are associated with endothelial injury and amplification of vascular inflammation [7,34]. These structures maintain a prooxidative and procoagulant environment, favoring endothelial dysfunction and microthrombosis [7].

Platelets

mCRP accumulates on activated platelets and is retained within platelet aggregates [36,67]. pCRP binds to activated platelet membranes and undergoes conformational dissociation into mCRP. This process depends on GPIIb-IIIa receptor activation and occurs under flow conditions [36,39]. The generation of mCRP on platelet surfaces links CRP conformational change to thromboinflammatory activity, as activated platelets provide a platform for local mCRP formation and retention [36,39]. mCRP has been shown to support platelet aggregation and may contribute to thrombus stabilization [39,67]. In obesity, chronic platelet activation increases the relevance of this mechanism for cardiovascular risk [7].

ROS and the Amplification Circuit

mCRP has been shown to increase ROS production in endothelial and immune cells in experimental models, thereby amplifying oxidative stress [33,34]. Oxidative stress promotes lipid membrane modification and creates conditions that favor local pCRP dissociation into mCRP on activated cells and inflammatory surfaces [14,36-38]. This supports a local amplification mechanism in which oxidative stress and mCRP formation are closely linked [14,33,34]. In obesity, persistent oxidative stress related to adipose tissue dysfunction, hypoxia, and metabolic imbalance further enhances this process [3,52,53].

Leptin

mCRP binds to the leptin receptor and interferes with its activation, thereby impairing leptin signaling [68,69]. At the molecular level, this interaction may disrupt intracellular signaling pathways associated with leptin receptor activation, contributing to reduced cellular responsiveness to leptin [68,69]. Experimental and clinical data support an association between this interaction and leptin resistance [68,69]. At the population level, the association between leptin, CRP, and cardiovascular risk markers supports the metabolic and vascular relevance of this mechanism [70,71].

Clinical and Laboratory Implications of mCRP

Conformational conversion generates an isoform with distinct biological properties, implicated in chronic tissue inflammation [14,37,72]. These effects are mediated through endothelial activation, increased expression of adhesion molecules, and enhanced leukocyte recruitment. In addition, mCRP interacts with platelets and immune cells, contributing to vascular inflammation and thrombosis.

Given that age is frequently associated with chronic low-grade inflammation, these observations may indicate a possible link to a persistent inflammatory status, although a direct correlation with inflammatory markers remains unclear [26-28,73]. mCRP may circulate bound to extracellular microparticles, allowing tissue distribution without proportional increases in total CRP [44]. Standard hsCRP assays quantify predominantly pCRP and do not distinguish conformational isoforms [74,75]. The development of analytical methods for mCRP detection supports its potential as a mechanistic biomarker of localized chronic inflammation [17,76,77].

Recent human studies have confirmed that circulating mCRP can be detected using Enzyme-Linked Immunosorbent Assay and mass spectrometry, and that it correlates with both clinical CRP levels and intratumoral mCRP abundance, suggesting its potential as a biomarker of active tissue inflammation [17]. Genetic variability influences CRP levels independently of inflammatory activity [78]. However, biological activity depends on local pCRP-to-mCRP conversion [14,37]. Standardization of analytical methods and the development of large prospective cohorts are essential steps in defining the clinical role of mCRP and integrating it into chronic inflammation assessment algorithms.

Emerging therapeutic strategies targeting the inhibition of pCRP dissociation into mCRP have been proposed. Small-molecule inhibitors, such as 1,6-bis(phosphocholine)-hexane, can block pCRP binding to activated cell membranes and prevent its conformational transition, while compounds such as C10M inhibit phosphocholine-dependent interactions and reduce downstream proinflammatory responses. This approach supports the concept that mCRP may serve not only as a biomarker but also as a potential therapeutic target in chronic inflammation and cardiovascular risk [79-81].

## Conclusions

Obesity creates a persistent proinflammatory environment characterized by hypoxia, oxidative stress, and endothelial activation that favors the local generation of biologically active forms of CRP. In this context, mCRP can be considered more than a simple biochemical epiphenomenon. It appears to act as a local mediator involved in maintaining tissue inflammation and amplifying the inflammatory response. Data from experimental models and small patient cohorts suggest that mCRP more accurately reflects active inflammation at the vascular and metabolic levels compared with total CRP or hsCRP. Determination of mCRP could contribute to a more accurate assessment of cardiometabolic risk and the identification of residual inflammation not detected by conventional biomarkers. However, clinical applicability is conditional on the standardization of detection methods and their validation in large prospective studies. Clarifying its prognostic and therapeutic role is an essential direction for future research in obesity-associated inflammation.

## References

[1] Saltiel AR, Olefsky JM (2017). Inflammatory mechanisms linking obesity and metabolic disease. J Clin Invest.

[2] Stafeev IS, Vorotnikov AV, Ratner EI, Menshikov MY, Parfyonova YV (2017). Latent inflammation and insulin resistance in adipose tissue. Int J Endocrinol.

[3] Rohm TV, Meier DT, Olefsky JM, Donath MY (2022). Inflammation in obesity, diabetes, and related disorders. Immunity.

[4] Iyer A, Fairlie DP, Prins JB, Hammock BD, Brown L (2010). Inflammatory lipid mediators in adipocyte function and obesity. Nat Rev Endocrinol.

[5] Arner P, Rydén M (2015). Fatty acids, obesity and insulin resistance. Obes Facts.

[6] Berg AH, Scherer PE (2005). Adipose tissue, inflammation, and cardiovascular disease. Circ Res.

[7] Wang Z, Nakayama T (2010). Inflammation, a link between obesity and cardiovascular disease. Mediators Inflamm.

[8] Rodríguez-Hernández H, Simental-Mendía LE, Rodríguez-Ramírez G, Reyes-Romero MA (2013). Obesity and inflammation: epidemiology, risk factors, and markers of inflammation. Int J Endocrinol.

[9] Kosovski IB, Ghiga D, Ciurea CN, Cucoranu DC, Demian L, Gliga FI, Bacârea A (2024). Evaluation of fasting glucose-insulin-C-peptide-derived metabolic indices for identifying metabolic syndrome in young, healthy adults. Nutrients.

[10] Bacârea A, Tarcea M, Boţianu PV, Ruţă F, Bacârea V (2015). Age cut-off for type 2 diabetes mellitus screening amongst young adults from Mures District, Romania--a pilot study. Obes Res Clin Pract.

[11] Kosovski IB, Bacârea V, Ghiga D, Ciurea CN, Cucoranu DC, Hutanu A, Bacârea A (2024). Exploring the link between inflammatory biomarkers and adipometrics in healthy young adults aged 20-35 years. Nutrients.

[12] Verma S, Szmitko PE, Yeh ET (2004). C-reactive protein: structure affects function. Circulation.

[13] Farì G, Ranieri M, Marvulli R (2023). Is there a new road to spinal cord injury rehabilitation? A case report about the effects of driving a Go-Kart on muscle spasticity. Diseases.

[14] Thiele JR, Habersberger J, Braig D (2014). Dissociation of pentameric to monomeric C-reactive protein localizes and aggravates inflammation: in vivo proof of a powerful proinflammatory mechanism and a new anti-inflammatory strategy. Circulation.

[15] Eisenhardt SU, Thiele JR, Bannasch H, Stark GB, Peter K (2009). C-reactive protein: how conformational changes influence inflammatory properties. Cell Cycle.

[16] Granata V, Pagani I, Morenghi E (2023). Modulation of NBAS-related functions in the early response to SARS-CoV-2 infection. Int J Mol Sci.

[17] Fuglestad AJ, Bousquet PA, Køstner AH (2025). Circulating levels of the proinflammatory monomeric isoform of C-reactive protein (mCRP) correlate with intra-tumoral mCRP abundance in stage II-III colon cancer patients. J Inflamm Res.

[18] Slevin M, Heidari N, Azamfirei L (2022). Monomeric C-reactive protein: current perspectives for utilization and inclusion as a prognostic indicator and therapeutic target. Front Immunol.

[19] Poddar NK, Khan A, Fatima F, Saxena A, Ghaley G, Khan S (2023). Association of mTOR pathway and conformational alterations in C-reactive protein in neurodegenerative diseases and infections. Cell Mol Neurobiol.

[20] Li Q, Xu W, Xue X (2016). Presence of multimeric isoforms of human C-reactive protein in tissues and blood. Mol Med Rep.

[21] Park J, Sohn JH, Han SM, Park YJ, Huh JY, Choe SS, Kim JB (2020). Adipocytes are the control tower that manages adipose tissue immunity by regulating lipid metabolism. Front Immunol.

[22] Braig D, Nero TL, Koch HG (2017). Transitional changes in the CRP structure lead to the exposure of proinflammatory binding sites. Nat Commun.

[23] Okemefuna AI, Stach L, Rana S, Buetas AJ, Gor J, Perkins SJ (2010). C-reactive protein exists in an NaCl concentration-dependent pentamer-decamer equilibrium in physiological buffer. J Biol Chem.

[24] Rizo-Téllez SA, Sekheri M, Filep JG (2023). C-reactive protein: a target for therapy to reduce inflammation. Front Immunol.

[25] Asztalos BF, Horan MS, Horvath KV, McDermott AY, Chalasani NP, Schaefer EJ (2014). Obesity associated molecular forms of C-reactive protein in human. PLoS One.

[26] Molins B, Peña E, de la Torre R, Badimon L (2011). Monomeric C-reactive protein is prothrombotic and dissociates from circulating pentameric C-reactive protein on adhered activated platelets under flow. Cardiovasc Res.

[27] Franceschi C, Garagnani P, Parini P, Giuliani C, Santoro A (2018). Inflammaging: a new immune-metabolic viewpoint for age-related diseases. Nat Rev Endocrinol.

[28] Ferrucci L, Corsi A, Lauretani F (2005). The origins of age-related proinflammatory state. Blood.

[29] Volanakis JE (2001). Human C-reactive protein: expression, structure, and function. Mol Immunol.

[30] Gabay C, Kushner I (1999). Acute-phase proteins and other systemic responses to inflammation. N Engl J Med.

[31] Black S, Kushner I, Samols D (2004). C-reactive protein. J Biol Chem.

[32] Pepys MB, Hirschfield GM (2003). C-reactive protein: a critical update. J Clin Invest.

[33] Schauber J, Dorschner RA, Yamasaki K, Brouha B, Gallo RL (2006). Control of the innate epithelial antimicrobial response is cell-type specific and dependent on relevant microenvironmental stimuli. Immunology.

[34] Khreiss T, József L, Potempa LA, Filep JG (2004). Conformational rearrangement in C-reactive protein is required for proinflammatory actions on human endothelial cells. Circulation.

[35] Zeinolabediny Y, Kumar S, Slevin M (2021). Monomeric C-reactive protein - a feature of inflammatory disease associated with cardiovascular pathophysiological complications?. In Vivo.

[36] Eisenhardt SU, Habersberger J, Murphy A (2009). Dissociation of pentameric to monomeric C-reactive protein on activated platelets localizes inflammation to atherosclerotic plaques. Circ Res.

[37] Sproston NR, Ashworth JJ (2018). Role of C-reactive protein at sites of inflammation and infection. Front Immunol.

[38] Rajab IM, Hart PC, Potempa LA (2020). How C-reactive protein structural isoforms with distinctive bioactivities affect disease progression. Front Immunol.

[39] Pezeshkpoor B, Zimmer N, Marquardt N (2013). Deep intronic 'mutations' cause hemophilia A: application of next generation sequencing in patients without detectable mutation in F8 cDNA. J Thromb Haemost.

[40] Martens MD, Holody CD, Wells L (2024). Reactive oxygen species modulator 1 plays an obligate role in cardiomyocyte hypertrophy. Circ Res.

[41] Fujita M, Takada YK, Izumiya Y, Takada Y (2014). Binding of monomeric C-reactive protein to integrins αvβ3 and α4β1 is related to its pro-inflammatory action. PLoS One.

[42] Olson ME, Hornick MG, Stefanski A (2023). A biofunctional review of C-reactive protein (CRP) as a mediator of inflammatory and immune responses: differentiating pentameric and modified CRP isoform effects. Front Immunol.

[43] Ahrens I, Domeij H, Eisenhardt SU (2011). Opposing effects of monomeric and pentameric C-reactive protein on endothelial progenitor cells. Basic Res Cardiol.

[44] Verwilligen RA, Mulder L, Rodenburg FJ, Van Dijke A, Hoekstra M, Bussmann J, Van Eck M (2022). Stabilin 1 and 2 are important regulators for cellular uptake of apolipoprotein B-containing lipoproteins in zebrafish. Atherosclerosis.

[45] Thompson D, Pepys MB, Wood SP (1999). The physiological structure of human C-reactive protein and its complex with phosphocholine. Structure.

[46] Salazar J, Martínez MS, Chávez-Castillo M (2014). C-reactive protein: an in-depth look into structure, function, and regulation. Int Sch Res Notices.

[47] Jabs WJ, Theissing E, Nitschke M (2003). Local generation of C-reactive protein in diseased coronary artery venous bypass grafts and normal vascular tissue. Circulation.

[48] Kaplan M, Hamoud S, Tendler Y, Meilin E, Lazarovitch A, Nitecki S, Hayek T (2014). A significant correlation between C-reactive protein levels in blood monocytes derived macrophages versus content in carotid atherosclerotic lesions. J Inflamm (Lond).

[49] Rull A, Camps J, Alonso-Villaverde C, Joven J (2010). Insulin resistance, inflammation, and obesity: role of monocyte chemoattractant protein-1 (or CCL2) in the regulation of metabolism. Mediators Inflamm.

[50] Kojta I, Chacińska M, Błachnio-Zabielska A (2020). Obesity, bioactive lipids, and adipose tissue inflammation in insulin resistance. Nutrients.

[51] de A Boleti AP, de O Cardoso PH, Frihling BEF, Silva PSE, de Moraes LF, Migliolo L (2023). Adipose tissue, systematic inflammation, and neurodegenerative diseases. Neural Regen Res.

[52] Wood IS, Trayhurn P (2008). Cellular hypoxia and adipose tissue dysfunction in obesity. Proc Nutr Soc.

[53] García-Sánchez A, Gámez-Nava JI, Díaz-de la Cruz EN (2020). The effect of visceral abdominal fat volume on oxidative stress and proinflammatory cytokines in subjects with normal weight, overweight and obesity. Diabetes Metab Syndr Obes.

[54] Zorena K, Jachimowicz-Duda O, Ślęzak D, Robakowska M, Mrugacz M (2020). Adipokines and obesity. Potential link to metabolic disorders and chronic complications. Int J Mol Sci.

[55] Vorotnikov AV, Stafeev IS, Menshikov MY, Shestakova MV, Parfyonova YV (2019). Latent inflammation and defect in adipocyte renewal as a mechanism of obesity-associated insulin resistance. Biochemistry (Mosc).

[56] Cani PD, Jordan BF (2018). Gut microbiota-mediated inflammation in obesity: a link with gastrointestinal cancer. Nat Rev Gastroenterol Hepatol.

[57] Shen J, Obin MS, Zhao L (2013). The gut microbiota, obesity and insulin resistance. Mol Aspects Med.

[58] Le Chatelier E, Nielsen T, Qin J (2013). Richness of human gut microbiome correlates with metabolic markers. Nature.

[59] Cani PD, Amar J, Iglesias MA (2007). Metabolic endotoxemia initiates obesity and insulin resistance. Diabetes.

[60] Turnbaugh PJ, Hamady M, Yatsunenko T (2009). A core gut microbiome in obese and lean twins. Nature.

[61] Potempa M, Hart PC, Rajab IM, Potempa LA (2025). Redefining CRP in tissue injury and repair: more than an acute pro-inflammatory mediator. Front Immunol.

[62] Tilg H, Moschen AR (2014). Microbiota and diabetes: an evolving relationship. Gut.

[63] Ouchi N, Kihara S, Funahashi T (2003). Reciprocal association of C-reactive protein with adiponectin in blood stream and adipose tissue. Circulation.

[64] Tchernof A, Nolan A, Sites CK, Ades PA, Poehlman ET (2002). Weight loss reduces C-reactive protein levels in obese postmenopausal women. Circulation.

[65] Stanimirovic J, Radovanovic J, Banjac K (2022). Role of C-reactive protein in diabetic inflammation. Mediators Inflamm.

[66] Melnikov I, Kozlov S, Okhota S (2024). Higher monomeric C-reactive protein levels are associated with premature coronary artery disease. Front Immunol.

[67] Slevin M, Matou-Nasri S, Turu M (2010). Modified C-reactive protein is expressed by stroke neovessels and is a potent activator of angiogenesis in vitro. Brain Pathol.

[68] Sudhakar M, Silambanan S, Chandran AS, Prabhakaran AA, Ramakrishnan R (2018). C-reactive protein (CRP) and leptin receptor in obesity: binding of monomeric CRP to leptin receptor. Front Immunol.

[69] Hribal ML, Fiorentino TV, Sesti G (2014). Role of C reactive protein (CRP) in leptin resistance. Curr Pharm Des.

[70] Martin SS, Qasim AN, Rader DJ, Reilly MP (2012). C-reactive protein modifies the association of plasma leptin with coronary calcium in asymptomatic overweight individuals. Obesity (Silver Spring).

[71] Romero-Corral A, Sierra-Johnson J, Lopez-Jimenez F (2008). Relationships between leptin and C-reactive protein with cardiovascular disease in the adult general population. Nat Clin Pract Cardiovasc Med.

[72] Tauziède-Espariat A, Beccaria K, Dangouloff-Ros V (2023). A comprehensive analysis of infantile central nervous system tumors to improve distinctive criteria for infant-type hemispheric glioma versus desmoplastic infantile ganglioglioma/astrocytoma. Brain Pathol.

[73] Song L, Tang Y, Law BY (2024). Targeting calcium signaling in Alzheimer's disease: challenges and promising therapeutic avenues. Neural Regen Res.

[74] Pohanka M (2022). Diagnoses based on C-reactive protein point-of-care tests. Biosensors (Basel).

[75] Coelho Graça D, Golaz O, Magnin JL, Fleurkens H, Rossier MF, Lescuyer P, Vuilleumier N (2018). CRP-based cardiovascular risk assessment: new conventional CRP assay fit for purpose?. J Appl Lab Med.

[76] Hornick MG, Potempa LA (2023). Monomeric C-reactive protein as a biomarker for major depressive disorder. Front Psychiatry.

[77] Almulla AF, Niu M, Stoyanov D, Zhang Y, Maes M (2025). Monomeric CRP and negative acute phase proteins, but not pentameric CRP, as biomarkers of major depression and MDMD. Acta Neuropsychiatr.

[78] Li X, Ploner A, Wang Y (2023). Rare functional variants in the CRP and G6PC genes modify the relationship between obesity and serum C-reactive protein in white British population. Mol Genet Genomic Med.

[79] Pepys MB, Hirschfield GM, Tennent GA (2006). Targeting C-reactive protein for the treatment of cardiovascular disease. Nature.

[80] Filep JG (2023). Targeting conformational changes in C-reactive protein to inhibit pro-inflammatory actions. EMBO Mol Med.

[81] Caprio V, Badimon L, Di Napoli M (2018). pCRP-mCRP dissociation mechanisms as potential targets for the development of small-molecule anti-inflammatory chemotherapeutics. Front Immunol.

